# Evaluating adherence in an active-controlled HIV pre-exposure prophylaxis trial (PrEPVacc) to inform the estimation of HIV incidence in a counterfactual placebo arm

**DOI:** 10.1080/25787489.2025.2513684

**Published:** 2025-06-11

**Authors:** Sheila Kansiime, Christian Holm Hansen, Henry Bern, Julie Fox, David Dunn, Eugene Ruzagira, Richard Hayes, Sheena Mc cormack

**Affiliations:** a Uganda Research Unit, Medical Research Council/ Uganda Virus Research Institute and London School of Hygiene and Tropical Medicine, Entebbe, Uganda; b Medical Research Council International Statistics and Epidemiology Group, London School of Hygiene and Tropical Medicine, London, UK; c Medical Research Council Clinical Trials Unit, london, UK; d King’s College London, London, UK

**Keywords:** Pre-exposure prophylaxis, counterfactual placebo hiV incidence, adherence-efficacy, non-inferiority pre-exposure prophylaxis trials, emtricitabine/ tenofovir

## Abstract

**Background:**

Inferring the counterfactual placebo HIV incidence using the estimated effectiveness of emtricitabine/tenofovir (TDF/FTC) in active-controlled pre-exposure prophylaxis (PrEP) trials has been suggested. however, it has not yet been widely applied. in this article, we evaluate adherence to TDF/FTC in the PrEPVacc trial (NCT04066881) and consider how such adherence data could be used to estimate the effectiveness of TDF/FTC and subsequently, HIV incidence in a counterfactual placebo arm in a predominantly female population.

**Methods:**

From December 2020 to March 2023 participants were recruited into the trial which included a comparison of emtricitabine/tenofovir alafenamide (TAF/FTC) to TDF/FTC as PrEP over 26 weeks of follow-up, in Uganda, Tanzania, and south Africa. PrEP adherence was assessed in various ways.

**Results:**

Of 697 participants dispensed TDF/FTC, 87% were female, 54% were ≥ 25 years, and 59% were sex workers. in a random sample (41%) assessed at visit 6 (week 8), 76% had detectable TFV-DP levels, with 22% reaching levels consistent with ≥2 pills/week. Males, Verulam and Mbeya participants, those ≥ 25 years, not single, subsistence fisheries workers, and those who had any STI at baseline were more likely to have higher adherence. Of those assessed at visit 6, 29% were identified as white coat dosing. estimated (crude) HIV incidence risk reduction ranged from 10% to 65%.

**Conclusions:**

TDF/FTC adherence in the PrePVacc trial was low, with considerable levels of white coat dosing. inferring the counterfactual placebo HIV incidence using the estimated effectiveness of TDF/FTC is a promising approach, however, the approach requires further elaboration and evaluation.

## Background

HIV pre-exposure prophylaxis (PrEP) is a highly efficacious preventive intervention when adhered to or taken consistently during periods of risk [1–3]. Accurate adherence measurement is particularly important for active-controlled HIV PrEP trials when TDF/FTC is the comparator arm, as adherence data and the known effectiveness of TDF/FTC can be used to infer an estimate of HIV incidence in a counterfactual placebo arm, i.e. incidence in the absence of drug, also referred to as a background HIV incidence rate [4]. This estimate is useful for approximating the true efficacy of novel products in comparison to no intervention [5–7].

Several adherence measurement approaches for oral PrEP have been used during the conduct of trials and in subsequent implementation projects. Some of these include using self-report data, pill counts, pill dispensing data, and pharmacological measures such as drug levels in single plasma samples, peripheral blood mononuclear cells (PBMCs), red blood cells collected through dried blood spots (DBS), hair, and urine [8–10]. No one approach can provide a comprehensive assessment of adherence informative for the evaluation of HIV prevention efficacy and effectiveness of oral PrEP [9,11]. Drug levels are accepted as the gold standard for adherence to a daily regimen but may under-estimate consistent ‘event-driven’ use at the time of risk, when risk is infrequent, and they are costly. In trials, drug level assessments are usually limited to a sub-group [3,5]. Self-report is inexpensive, and most informative with respect to consistent use around condomless sex, however, it can be affected by recall and social desirability biases [12]. Short term adherence measures (such as urine tenofovir tests measuring adherence in the last 2 days and plasma tenofovir concentration measurements) are less costly but are susceptible to ‘white coat dosing’ whereby participants are more likely to take drug in anticipation of an upcoming clinic visit, especially when conducted at pre-scheduled clinic visits. Also, they have not generally been used in HIV prevention trials.

Accuracy of the different adherence assessment approaches could vary between study populations [12]. For example, accuracy of self-report and white coat dosing effect on short-term pharmacological measures, are expected to vary by population, questions asked, experience of study staff, and frequency of data collection.

In 2015, the World Health Organization (WHO) recognised oral TDF/FTC as the standard of care for HIV prevention trials. As a result, subsequent HIV PrEP trials have had to be designed as active-controlled studies. Unfortunately, this tends to require exceptionally large sample sizes especially when intending to assess non-inferiority to a highly effective intervention [13]. Alternatively, there is increasing acceptance of the use of counterfactual placebo incidence estimates. One of the more promising approaches to estimating counterfactual placebo incidence, is utilising the known effectiveness of TDF/FTC and the observed adherence data in a trial.

Several articles have suggested how Tenofovir-Diphosphate (TFV-DP) levels estimating the average number of pills taken by a participant per week over the last 6 weeks, can be utilised to infer expected risk reduction [4,6, 14–16]. Confidence intervals (CIs) for the estimated risk reduction attributed to a given level of adherence are wide and more studies are needed to increase precision [16]. This is a key limitation of this counterfactual placebo incidence estimation approach. Additionally, there is limited information on the use of this approach among females. Disparate results from TDF/FTC placebo-controlled effectiveness trials among females add to the complexity [13,16,17]. Furthermore, it is unclear how ‘event-driven’ dosing can be incorporated in these analyses. Individuals who practice ‘event-driven’ dosing may have greater risk reduction at low levels of overall drug than those who do not. Similarly, those practising white coat dosing may get less protection despite having detectable levels of drug.

Alongside measuring adherence, it is key to understand who is more likely to adhere. Higher PrEP adherence among individuals considered to be at higher risk of HIV based on location, demographic characteristics or other HIV risk indicators would be expected to have a higher impact on HIV incidence than higher adherence among individuals who are considered to be at lower risk. Some HIV prevention trials found higher adherence among individuals at higher risk, but others did not [4].

To enable comprehensive interpretation of adherence data in PrEP trials, it is important that data from various adherence measures are utilised to gain a comprehensive understanding of adherence, and that factors influencing adherence are understood. Using data from a recently completed HIV prophylactic vaccine and PrEP trial, PrEPVacc (NCT04066881), we aimed to describe adherence to TDF/FTC among the study participants using various adherence measures, assess demographic and risk behaviour characteristics associated with adherence, assess the reliability of other adherence measures in comparison to drug levels in red blood cells and investigate white coat dosing. We then considered how such adherence data could be used to inform an estimate of HIV incidence in a counterfactual placebo arm utilising data at group level and individual level.

## Methods

### Study setting and design

The PrEPVacc trial was conducted at four study sites in three countries. The trial target population included female bar workers and female sex workers (FSW) in Dar es Salaam and Mbeya, Tanzania; the general population in areas of known high HIV incidence in Durban-Verulam, South Africa; FSW, female and male fisher folk in Masaka, Uganda.

(Fisher folk in Uganda also referred to as subsistence fisheries workers are considered to be a group at higher risk of HIV [18–20]. They include fisher men, fishmongers, fish processors, boat makers, repairers, etc., and shopkeepers, bar/restaurant/lodge owners, and commercial sex workers in fishing communities).

**Figure 1:**
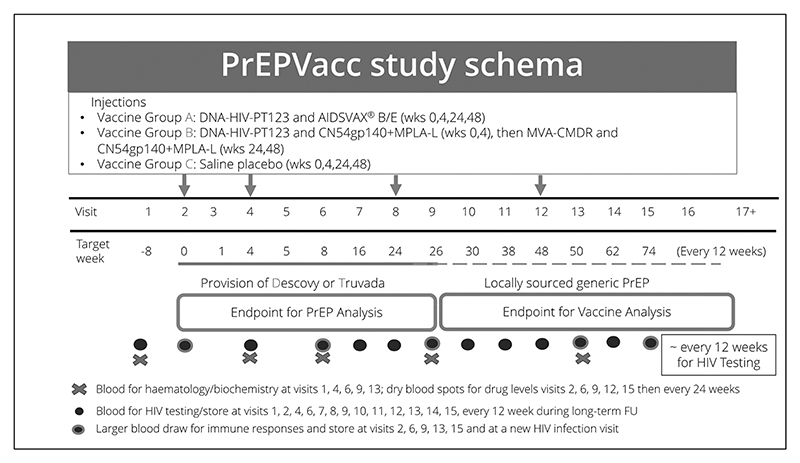
PrEPVacc study schema.

The PrEPVacc trial was a randomised (1:1:1) phase IIb HIV vaccine trial evaluating the safety and efficacy of two combination HIV vaccine regimens, i.e. HIV DNA + gp120/alum and HIV DNA, MVA-CMDR + gp140/MPLA, each compared to placebo, with a concurrent randomisation (1:1) to compare emtricitabine (FTC)/Tenofovir alafenamide (TAF) to tenofovir disoproxil (TDF)/emtricitabine (FTC) as PrEP for a target of 26 weeks. Beyond week 26, participants were referred to local providers for continued access to TDF/FTC-based PrEP. The vaccines were administered at four time points: target weeks 0, 4, 24, and 48 (Figure 1). Overall, target follow-up for the trial was a minimum of 74 weeks from enrolment (Figure 1). Details can be found elsewhere [21–23].

The primary endpoint for the analysis of both the PrEP interventions and vaccines is confirmed incident HIV infection. Infections diagnosed at or before visit 9 (2 weeks after the third trial vaccination) contribute to the primary PrEP analysis, while infections occurring thereafter contribute to the primary vaccine analyses (Figure 1). HIV testing followed a trial-specific HIV testing algorithm, utilising Bio-Rad Genscreen™ ULTRA HIV Ag-Ab assay for initial screening. Positive results were confirmed using the HIV-1 RNA PCR assay.

The PrEP component aimed to show that the effectiveness of TAF/FTC was not unacceptably lower than the effectiveness of TDF/FTC through analyses incorporating a counterfactual placebo HIV incidence rate. The analyses presented in this article focus on measuring adherence in the TDF/FTC arm during the active-controlled PrEP trial component, which could then be used to infer the counterfactual placebo HIV incidence rate for the active-controlled PrEP trial component based on the known effectiveness of TDF/FTC at different levels of adherence.

Participants were considered eligible for the trial if they were HIV-uninfected adults aged 18–40 years at the time of enrolment, willing and able to provide informed consent, willing to receive promotion of PrEP, willing and able to comply with the visit schedule and provide blood, urine and other samples, available for at least 82 weeks from screening and, if female, willing to use a highly effective method of contraception [22]. A comprehensive list of inclusion and exclusion criteria can be found elsewhere [22]. For participants to be enrolled in the PrEPVacc trial they needed to be willing to receive promotion of PrEP. For inclusion in the PrEP trial analyses, they needed to accept at least one dispensing of drug and attend at least one follow up visit with an HIV test.

### PrEP dispensing and adherence assessment

Participants were advised to take one PrEP pill daily, and also informed of its effectiveness when taken around the times of sexual activity. PrEP was primarily dispensed at four study visits: 60 tablets at enrolment (visit 2, target week 0); 90 tablets at visit 4 (target week 4); at visit 7 (target week 16), tablet reconciliation was conducted, participants were provided with sufficient tablets to last until visit 9 (week 26, two weeks after the third vaccination), typically 30, 60, or 90 tablets. Dispensing at visit 8 (target week 24) s conducted if needed to provide sufficient PrEP to last until visit 9. Dispensing at other visits up to until visit 9 was conducted if needed, for example in cases of lost PrEP, or missed dispensing visits. Post visit 9, participants were encouraged to continue taking PrEP and referred to a local PrEP provider.

PrEP adherence was measured for all participants using self-reported adherence during a face- to- face interview, pill dispensing data, and UrSure rapid urine Tenofovir tests [24]. In a random subset of participants, DBS were tested for drug levels in red blood cells at visit 6 (target week 8), and retested for a smaller subset at visit 9 (target week 26). The adherence assessment measures used in the trial are described in more detail in Table 1.

### Statistical analyses

Data management and analysis were conducted in Open Clinica (Community Edition) and Stata version 18.0 (College Station, TX), respectively. The analysis population comprises all participants randomised to TDF/FTC who were HIV negative at enrolment, ever dispensed PrEP, and attended at least one follow up visit with an HIV test in the PrEP component. Participants in the TAF/FTC arm were not included in this analysis.

### PrEP adherence and factors associated

Adherence estimates based on the different measures were presented. Univariable and multivariable ordinal logistic regression models were used to assess associations with higher levels of adherence based on TFV-DP levels in DBS. Potential factors were identified from literature and within the trial database. At the multivariable analysis, forward stepwise model building was utilised, with predictors added to the model if they had a *p* value < 0.2. Site and sex were included in the multivariable model a priori.

Once the final multivariable model was determined, predictors that had been excluded from the multivariable model were re-introduced one at a time, and their adjusted ORs and multivariable *p* values estimated.

### Assessing reliability of other adherence measures in comparison to DBS

Reliability was assessed in two ways:

- Associations: We assessed association between the overall estimate of adherence (described in Table 1) for a given measure of adherence and TFV-DP levels at visit 6 using ordinal logistic regression, adjusting for other factors associated with adherence as measured on DBS.
- Accuracy assessments: Direct comparisons were made between visit 6 urine tenofovir results, Emtricitabine triphosphate (FTC-TP) levels, and separately TFV-DP levels. Comparisons with TFV-DP levels indicated how informative urine tests were for long-term adherence. For self-report, visit 6 self-reported data on adherence around the most recent condomless sex act was compared with visit 6 TFV-DP and FTC-TP results taking into consideration days since last condomless sex act at visit 6, and (separately) median days since most recent condomless sex acts assessed at visits 4, 6, 7, 8, and 9.

### White coat dosing and associated factors

White coat dosing was defined as a participant having TFV-DP < 350 fmol/punch (averaging less than 2 pills per week) and FTC-TP > 0.1 pmol/punch (recent dose in the last 48 h) [10,25]. Percentages of participants with evidence of white coat dosing were reported overall and in subgroups. Factors associated with white coat dosing, were investigated using univariable and multi-variable Logistic regression models and a modelling approach similar to that described for factors associated with adherence above.

### Missing data

Missing data have been reported in the results section. Additionally, for overall estimates of adherence across the various measures, if a participant had a missing result (e.g. on self-reported pill use or urine test at a given visit), their available results from other visits were used to estimate an average result across all expected assessments. If the participant did not have any results at all, they were excluded from the analysis.

## Results

### Participant demographics and overall adherence

Overall adherence is presented in Tables 2 and 3. Of 697 participants dispensed TDF/FTC, 87% were female, 54% were ≥ 25 years, 40% were single, 59% were sex workers, 32% were from Mbeya, 30% from Masaka, 26% from Verulam, and 13% from Dar es Salaam (Table 3). There was close similarity between all study participants and participants whose DBS were tested for drug levels in red blood cells at visit 6 (week 8) (Table 3).

At visit 6, 76% of the 290 participants assessed had detectable TFV-DP levels of drug; 54% had TFV-DP levels equivalent to <2 tablets per week but detectable; 14% had TFV-DP levels equivalent to 2–3 tablets per week, and 8% had TFV-DP levels equivalent to 4 or more tablets per week (Table 2). At the same visit, 69% of participants had detectable Emtricitabine triphosphate (FTC-TP) levels (estimating adherence over the last 2–4 d), 19% had detectable but unquantifiable levels of drug, and 49% had quantifiable levels of drug (>0.1 pmol/punch) (Table 2).

At visit 9 (week 26), 51% of the 39 participants assessed had detectable TFV-DP levels of drug; 38% had TFV-DP levels equivalent to <2 tablets per week but detectable; 13% had TFV-DP levels equivalent to 2–3 tablets per week, and 0% had TFV-DP levels equivalent to 4 or more tablets per week.

Regarding the other measures of adherence: 88% of participants reported taking PrEP before at least 3 of 5 assessed sex acts; 89% had a medicine possession ratio greater or equal to 75% at visit 9, and 74% had drug presence confirmed in at least 2 out of 3 UrSure rapid urine Tenofovir tests conducted (Table 2).

### Factors associated with adherence as measured by DBS at visit 6

The Verulam site had the highest adherence with 94% of its study participants having detectable levels of TFV-DP at visit 6. At this site, approximately 50% and 28% of male and female participants, respectively, had adherence levels equivalent to ≥ 2 pills/week. At the Masaka site, 64% of study participants had detectable TFV-DP levels, with 44% male participants and 19% of female participants achieving adherence levels ≥2 pills/week. In Mbeya and Dar es Salaam, 77% and 68% of participants, respectively, had detectable levels of TFV-DP levels, while levels ≥ 2 pills/week were observed in 15% and 11% of participants, respectively.

**Figure 2:**
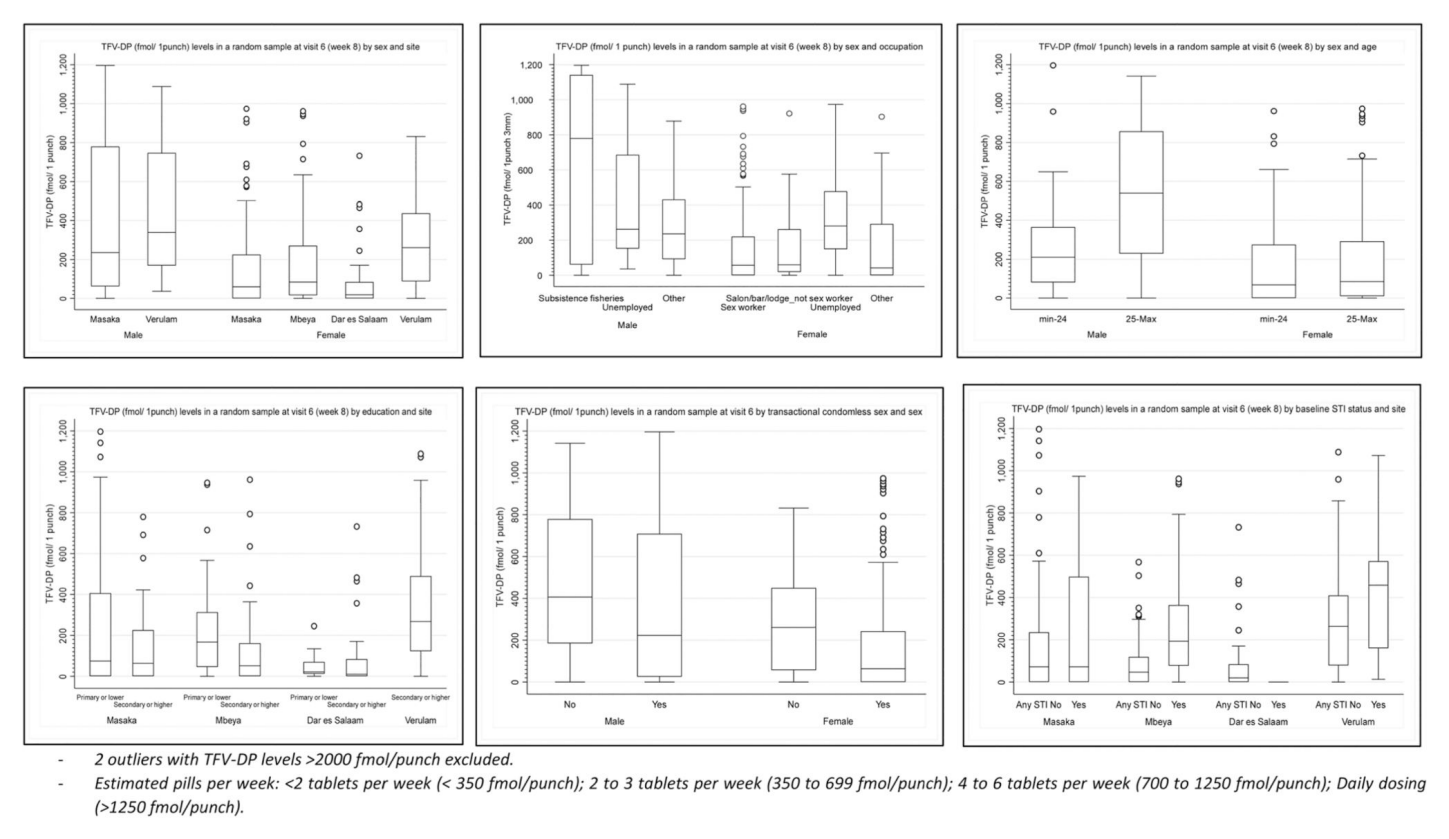
Adherence to TDF/FTC in the PrEPVacc PrEP trial as per TFV-DP levels in DBS. - 2 outliers with TFV-DP levels >2000 fmol/punch excluded. - estimated pills per week: <2 tablets per week (< 350 fmol/punch); 2–3 tablets per week (350–699 fmol/punch); 4–6 tablets per week (700–1250 fmol/punch); Daily dosing (>1250 fmol/punch).

Univariable analysis results have been presented in Table 3. Figure 2 shows TFV-DP levels in DBS at visit 6 by study site, sex, age group, baseline STI status, transactional condomless sex, and marital status. At multivariable analysis, women were less likely than men to have higher adherence, aOR (95% CI): 0.29 (0.12 − 0.70) (Table 3). Participants aged 25 years or older were more likely to have higher adherence than those who were younger, aOR (95% CI): 1.53 (0.93 − 2.51). In comparison to single participants, divorced/separated/widowed participants were more likely to have higher adherence, aOR (95% CI): 1.84 (0.92 − 3.70). Those who had any STI at baseline were more likely to have higher adherence than those who did not, aOR (95% CI): 2.46 (1.41 − 4.28). Subsistence fisheries workers were more likely to have higher adherence than sex workers, aOR (95% CI): 10.78 (1.76 − 66.12). Individuals with secondary education or higher were less likely to adhere, aOR (95% CI): 0.49 (0.28 − 0.86). This was unexpected and not reflected in the site-specific analyses (Figure 2). There was weak evidence of associations with reported risk indicators (Table 3).

### Assessing reliability of other adherence measures

Self-report on adherence around condomless sex acts, the medicine possession ratio at visit 9, and UrSure rapid urine Tenofovir tests results, demonstrated strong associations with TFV-DP levels (Supplementary Table 1a). However, only UrSure rapid urine tests reliably predicted detectable or undetectable levels of drug as per FTC-TP levels measuring adherence in the last 2–4 d with sensitivity: 95%; specificity: 86%. positive predictive value (PPV): 94%; negative predictive value: 89% (Supplementary Table 1b). The other measures did not predict TFV-DP or FTC-TP levels reliably enough to allow direct inference (Supplementary Tables 1c and 1d).

### Assessing white coat dosing

A total of 85 participants (29% of all participants; 59% of all with quantifiable levels of FTC-TP; 37% of all with TFV-DP <350 fmol/punch) met the definition of white coat dosing: TFV-DP <350 fmol/punch and FTC-TP > 0.1 pmol/punch [21] (Table 4; Supplementary Table 2).

Univariable analysis results have been presented in Table 4. At multivariable analysis, Verulam and Mbeya site participants were more likely to white coat dose than Masaka participants, aOR (95% CI): 3.08 (0.74 − 12.87) and 2.33 (0.99 − 5.48), respectively. Dar es Salaam participants were less likely than Masaka participants to white coat dose, aOR (95% CI): 0.57 (0.17–1.90). Muslim/other participants were more likely to white coat dose than Christians, aOR (95% CI): 3.52 (1.46 − 8.47). Occupation was strongly associated with white coat dosing (*p* = 0.037) with sex workers and those from ‘other’ occupations less likely to white coat dose. Participants who reported engaging in sex while drunk within the past 3 months were more likely to white coat dose than those who did not, aOR (95% CI): 1.82 (1.00 − 3.30). There was weak evidence suggesting that participants who had an STI at baseline were less likely to white coat dose than those who did not, aOR (95% CI): 0.70 (0.36 − 1.36) (Table 4).

### Considerations for using PrEP adherence data to estimate HIV incidence in a counterfactual placebo arm

- At group level: An overall counterfactual placebo incidence rate can be estimated by considering what the incidence rate would have been if there were no HIV incidence reduction attributable to the overall observed level of adherence to TDF/FTC. The observed categorised adherence estimate (i.e. in a trial measured using TFV-DP levels) can be used to estimate the expected risk reduction with reference to literature [14,26] (Table 5). The overall estimated risk reduction in a trial can be calculated as a weighted average of the adherence category-specific risk reduction effects. A crude counterfactual placebo incidence estimate can then be obtained as:
  $$\text{Counterfactual}\;\text{placebo}\;\text{incidence}=\frac{\;\text{Observed}\;\text{incidence}\;\text{in}\;\text{the}\;\text{TDF}\;\text{/}\;\text{FTC}\;\text{arm}\;}{\left(1-\;\text{estimated}\;\text{risk}\;\text{reduction}\;\right)}$$

- where feasible, the impact of potential confounding factors for the association between adherence and HIV incidence can partially be mitigated by conducting stratified analyses by levels of potential confounders, e.g. study site, sex, age category, etc.
- At individual level: If the sample size were large enough, and an estimate of each participant’s adherence were available (observed based on DBS data for each individual or otherwise imputed based on factors associated with adherence in the dataset), the adherence-efficacy estimate (obtained from literature for a participant’s level of adherence) could be incorporated as an offset variable (ln RR if on the log scale) in a Poisson model for HIV incidence in the TDF/FTC arm. Parameters (regression coefficients) for other relevant predictors of HIV incidence in the TDF/FTC arm would then be estimated (Equation (1)). Thereafter, a counterfactual estimate for each participant could be reached by using the fitted regression model but with the adherence efficacy offset removed (Equation (2)). CIs would then be obtained, and other inferences made. This approach would address concerns of confounding factors for the association between adherence and HIV incidence. However, it is limited by how well the adherence of each participant can be estimated, and the precision of the estimated HIV incidence rate in the TDF/FTC arm, which is dependent on the number of HIV infections observed. With fewer observed HIV infections, exact CIs and exact Poisson regression may be more appropriate.

(1) $$\text{ln}\;{\text{λ}}_{\text{i}}\;\text{=}\;{\text{β}}_{\text{0}}\;\text{+}\;{\text{β}}_{1}{\text{X}}_{\text{1i}}\;\text{+}\;{\text{β}}_{\text{2}}{\text{X}}_{\text{2i}}\;\ldots \;{\text{β}}_{\text{J}}{\text{X}}_{\text{Ji}}\;+\;\ln(\text{RR}i)$$

(2) $$\text{ln}\;{\text{Cλ}}_{\text{i}}={\text{β}}_{\text{0}}\;\text{+}\;{\text{β}}_{\text{1}}{\text{X}}_{\text{1i}}\;\text{+}\;{\text{β}}_{\text{2}}{\text{X}}_{\text{2i}}\;\ldots \;{\text{β}}_{\text{J}}{\text{X}}_{\text{Ji}}$$

where λ_i_ is the HIV incidence rate of an individual in the TDF/FTC arm

β_0_ is the ln of the HIV incidence rate when all predictors are zero (baseline).

β is the change in ln of the HIV incidence rate associated with a unit increase for a given covariate.

X is a covariate (e.g. study site, gender, age, etc.) in the model

J is the total number of covariates in the model

*i* indexes each individual contributing to this analysis

*RR* is the rate ratio attributed to a given level of adherence, i.e. (1 − estimated risk reduction).

Cλ_i_ is the counterfactual placebo HIV incidence rate of an individual in the TDF/FTC arm.

(When individual level PrEP adherence data are available at multiple time points in the trial, these analyses can be split by the time-period covered by an estimated level of adherence (e.g. using the Stata stsplit command), creating multiple rows of data for each participant, with each row having an estimate of PrEP adherence (and estimated risk reduction), an estimate of HIV incidence, and subsequently a counterfactual placebo incidence rate. The overall estimated counterfactual placebo HIV incidence rate of an individual would be a weighted average by person-years accrued in each of the time periods).

The estimated counterfactual placebo incidence rate will likely still be imprecise and need consideration of estimates from other counterfactual placebo incidence estimation approaches. PrEPVacc trial analyses utilising adherence data to inform a counterfactual placebo HIV incidence estimate will be incorporated in a subsequent article reconciling counterfactual placebo incidence estimates from other approaches (a pre-trial registration cohort and a post-PrEP component trial data set).

## Discussion

The majority (76%) of participants in the PrEPVacc PrEP trial had detectable TFV-DP levels at visit 6 (week 8). However, only 22% reached levels consistent with 2 or more pills per week. By visit 9 (week 26), approximately, 51% of participants had detectable TFV-DP levels, while 13% had levels consistent with taking 2 or more pills per week. A systematic review of placebo-controlled TDF/FTC trials among females highlighted that in the three trials that demonstrated a protective effect (Partners PrEP Study, TDF2 study in Botswana, and Bangkok Tenofovir Study), Tenofovir was detected in 67–83% of samples from a random subset of participants, compared with 24–30% in the two trials that had null results (FEM-PrEP, VOICE). This would suggest that the adherence observed in our trial may support an informative non-inferiority trial with participants receiving HIV risk reduction from TDF/FTC. However, the proportion of participants reaching drug levels consistent with 2 or more pills per week in our study was low compared to trials largely conducted among males at high risk of HIV, where 72–93% achieved these levels [27,28].

In our study, 47% and 18% of males and females, respectively, had adherence consistent with taking of 2 or more pills per week. The lower adherence among females in comparison to males is well documented but still surprising [17,29]. It might be expected that women would be more willing to take PrEP given their limited autonomy in managing HIV exposure risks in certain settings [30–33]. A direct dosing study among Kenyan women [34], demonstrated comparable levels of TFV-DP levels for women to those that have been published for men at the same number of pills taken per week [10], suggesting that, the observed disparity in our study reflects a genuine difference in the number of pills taken. Higher adherence among men was also noticeable in the other adherence measures used in the trial. Investigations into socio-behavioural drivers of this imbalance should increase clarity. In our study, subsistence fisheries workers (mostly men from Masaka) had higher levels of adherence than women in the same setting (Figure 2). This could be as a result of higher risk perception among subsistence fisheries workers [18,35]. (The small number of subsistence fisheries workers assessed limited comprehensive analyses).

Other demographic characteristics were also strongly associated with adherence. Verulam and Mbeya site participants, those aged 25 years or older, individuals who were divorced, separated, widowed, and those with any STI at baseline were more likely to have higher adherence. In our study, there was weak evidence of negative associations with self-reported HIV risk indicators. To increase accuracy, these predictors should ideally be taken into consideration when inferring the counterfactual placebo HIV incidence.

There were strong associations between the various adherence measures used in the study and drug levels measured in DBS. The UrSure urine Tenofovir tests used in the study reliably predicted detectable or undetectable Emtricitabine triphosphate (FTC-TP) levels reflecting adherence in the last 2–4 d. Based on these findings, we would strongly recommend using UrSure urine Tenofovir test to measure short-term adherence in HIV prevention studies.

However, high levels of white coat dosing were observed in our trial. This implied that despite having conducted multiple urine tenofovir tests, we were unable to reliably infer what the long-term adherence in the trial was until TFV-DP data from DBS were available. The combined data from the urine tenofovir tests, gave a higher estimate of overall adherence than the TFV-DP levels that measure average adherence over the last 6 weeks. Additionally, despite strong associations, self-reported adherence data around the last condomless sex acts and medicine possession ratio could not be used to reliably infer long-term adherence according to TFV-DP levels. This was somewhat expected, considering the recall and social desirability biases acknowledged by many [36,37].

Lastly, we considered how the adherence data in this trial could be utilised to estimate HIV incidence in a counterfactual placebo arm. The wide CIs of the expected risk reduction from the literature, for adherence levels less than 2 pills per week (Table 5) introduce considerable uncertainty. A trial with higher levels of adherence would be better suited for the adherence – efficacy approach to estimating a counterfactual placebo HIV incidence. More so, in order to adjust for confounders of the association between HIV incidence and adherence at analysis, a substantial number of sero-conversions need to be observed in the TDF/FTC arm of the active-controlled PrEP trial.

This analysis had multiple strengths. First, it utilised data from an HIV prevention trial that were collected, and cleaned rigorously, ensuring high data quality. Second, urine tenofovir tests have largely not been used in HIV prevention trials [25], increasing the originality of the results presented in this article. Third, the rapidly changing landscape in HIV prevention, implies that data on adherence to TDF/FTC from previous trials have largely not been interpreted with the same intention as ours, that is, to estimate a counterfactual placebo incidence rate, yet this will be crucial for future trials that will evaluate the non-inferiority of novel interventions compared with TDF/FTC.

This analysis also had several limitations. First, there were time periods during the conduct of the trial, when the UrSure Tenofovir urine tests kits were out of stock. During this time, urine samples were stored at −80° and tested once the kits were available. Urine tests were more likely to show no drug when performed on previously frozen samples as opposed to fresh samples (OR (95% CI): 3.0 (2.7–3.7)). We did not exclude or correct results from frozen urine samples in our analyses. We expect that this led to a slight underestimate of adherence in our study. Second, as is expected with self-reported data, there were notable inconsistencies highlighted when the same aspect was questioned in varying ways. For example, comparisons between the number of days since the last PrEP dose and adherence data around the last recent condomless sex act resulted in the identification of inconsistencies. To address this, study sites were asked to correct the data by referencing a detailed PrEP adherence diary card that was completed by each study participant. However, this measure could not resolve all the inconsistencies, as the PrEP diary cards were sometimes not completed correctly and comprehensively.

In reference to the observed adherence data in the PrEPVacc trial, this article describes how such data could be used to infer counterfactual placebo HIV incidence. Given the ease of collecting adherence data in trials, inferring the counterfactual placebo incidence using the effectiveness of TDF/FTC is likely to continue being used in future active-controlled trials. However, it is important that the methods for applying this approach are clearly defined, with attention paid to the imprecision of risk reduction estimates (especially when adherence is low), potential confounding factors for the association of adherence and HIV incidence, white coat dosing, and the potential impact of ‘event-driven’ adherence. Unless these outstanding concerns are resolved, this approach could lead to invalid or misleading results.

## Supplementary Material

Table 1a,1b,1c,1d and Table 2

## Figures and Tables

**Table 1:** Adherence assessment measures, time points, and analysis.

Adherence measure	Assessment time points	Analysis	Adherence categories
Self-reported adherence (before last condomless sex act)	Visit 4 (Target week 4) Visit 6 (Target week 8) Visit 7 (Target week 16) Visit 8 (Target week 24) Visit 9 (Target week 26)	(Target: All participants assessed; asked about the most recent condomless sex act before a visit) Participants were grouped into categories representing the total number of reported condomless sex acts where a PrEP tablet was reported taken before the sex act. PrEP adherence before a sex act was assumed to reflect adherence around the sex act.	(Of 5 sex acts) − 0 sex acts protected (or 0%). − 1-2 sex acts protected (or >0% but <41%) − 3-4 sex acts protected (or >40% but <81%) − 5 sex acts protected (or >80%)
Pill dispensing data (∼26 weeks adherence)	Visit 9 (Target week 26)	(Target: All participants assessed) Participants were categorised into a two-level variable using the medicine possession ratio. Medicine possession ratio (MPR)= (Total number of pills dispensed*100)/Number of days until visit 9. The medicine possession ratio was computed for participants once at visit 9. Tablets were dispensed primarily at visits 2, 4, 7, and 8 (if needed).	- MPR <75% - MPR ≥75%
UrSure rapid urine Tenofovir test results (last 2 d adherence)	Visit 4 (Target week 4) Visit 6 (Target week 8) Visit 7 (Target week 16)	(Target: All participants assessed) Participants were grouped into categories representing the proportion of urine tests that were indicative of drug presence.	− 0 tests (or 0%) − 1 test (or >0% but <34%) − 2 tests (or >33% but < 68%) − 3 tests (or > 67%)
Tenofovir-Diphosphate (TFV-DP) levels in RBCs (assessed using DBS) (last 6 weeks’ adherence)	Visit 6 (Target week 8) Visit 9 (Target week 26)	(Target: A random sample (41%) at visit 6; a smaller subset (*n* = 39) at visit 9) Based on analyses in the iPrEx OLE study [14, 10, 26], participants were grouped into categories reflecting estimated number of tablets taken per week in the preceding 6 wk. 4 or more tablets a week are expected to give sufficient (∼≥90%) protection against infection. Lower levels can also provide protection, especially if used in an ‘event-driven’ regimen.	- < 350 fmol/punch ∼ (<2 tablets per week) − 350 to 699 fmol/punch∼ (2 to 3 tablets per week) − 700 to 1250 fmol/punch∼ (4 to 6 tablets per week) - >1250 fmol/punch∼ (daily dosing).
Emtricitabine triphosphate (FTC-TP) levels in RBCs (assessed using DBS) (last 2−4 d’ adherence)	Visit 6 (Target week 8) Visit 9 (Target week 26)	(Target: A random sample (41%) at visit 6; a smaller subset (*n* = 39) at visit 9) Participants were grouped into categories reflecting whether they had detectable levels of drug or not.	- Undetectable - Detectable but unquantifiable - Quantifiable (>0.1 pmol/ punch)

**Table 2:** Adherence to TDF/FTC among participants in the PrEPVacc trial (PrEP component).

	Masaka (*N* = 213) *n* (%)	Mbeya (*N* = 216) *n* (%)	Dar es Salaam (*N* = 88) *n* (%)	Verulam (*N* = 180) *n* (%)	Overall (697) *n* (%)
Estimated number of pills per week (TFV-DP levels in DBS at visit 6)					
Not included in the random sample tested*	125	122	50	110	407
Undetectable	32 (36)	22 (23)	12 (32)	4 (6)	70 (24)
<2 tablets per week (< 350 fmol/punch)	35 (40)	58 (62)	22 (58)	42 (60)	157 (54)
2−3 tablets per week (350-699 fmol/punch)	12 (14)	8 (9)	3 (8)	18 (26)	41 (14)
4−6 tablets per week (700-1250 fmol/punch)	8 (9)	5 (5)	1 (3)	6 (9)	20 (7)
Daily dosing (>1250 fmol/punch)	1 (1)	1 (1)	0 (0)	0 (0)	2 (1)
Last 2−4 d adherence (FTC-TP levels in DBS at visit 6)					
Undetectable	49 (56)	28 (30)	9 (24)	5 (7)	91 (31)
Detectable but unquantifiable	3 (3)	28 (30)	16 (42)	9 (13)	56 (19)
Quantifiable (>0.1 pmol/punch)	36 (41)	38 (40)	13 (34)	56 (80)	143 (49)
Self-reported adherence (before the last condomless sex act)_of 5 sex acts**					
No sex acts data on file (N/A)	0 (0)	0 (0)	0 (0)	0 (0)	0 (0)
0 sex acts protected (or 0%)	28 (13)	3 (1)	4 (5)	1 (1)	36 (5)
1−2 sex acts protected (or >0% but <41%)	29 (14)	11 (5)	4 (5)	4 (2)	48 (7)
3−4 sex acts protected (or >40% but <81%)	79 (37)	52 (24)	23 (26)	62 (34)	216 (31)
5 sex acts protected (or >80%) Pill dispensing (medicine possession ratio at visit 9 (week 26))	77 (36)	150 (69)	57 (65)	113 (63)	397 (57)
MPR < 75%	44 (21)	18 (8)	5 (6)	8 (4)	75 (11)
MPR ≥ 75%	169 (79)	198 (92)	83 (94)	172 (96)	622 (89)
Urine tests indicative of drug (last 2 d adherence)of 3 urine tests**					
No urine test results on file (N/A)	3 (1)	0 (0)	0 (0)	1 (0)	4 (1)
0 tests (or 0%)	78 (37)	25 (12)	2 (2)	1 (1)	106 (15)
1 test (or >0% but <34%)	33 (16)	23 (11)	6 (7)	7 (4)	69 (10)
2 tests (or >33% but < 68%)	44 (21)	59 (27)	20 (23)	41 (23)	164 (24)
3 tests (or > 67%)	55 (26)	109 (50)	60 (68)	130 (72)	354 (51)

* Representativeness of the random sample for DBS testing is included in Table 3.

** Self report assessments were conducted at visits 4, 6, 7, 8, and 9; urine Tenofovir tests were conducted at visits 4, 6, and 7

**Table 3:** Factors associated with adherence to TDF/FTC as per dried blood spot results.

Socio-demographic variables	Categories	All study participants		Participants with DBS results		As per TFV DP levels in RBCs (assessed using DBS) (last 6 weeks adherence)^¥^				
		*n* (%)*		*n* (%)*		N (%) with adherence ≥ 2 pills/weeks	OR^§^ (95% CI)	*p* Value	aOR^§^ (95% CI)	*p* Value
		697 (100)		290 (41)		63 (22)				
Site	Masaka	213 (31)		88 (30)		21 (24)	Ref	*p* < 0.001	Ref	0.288
	Mbeya	216 (31)		94 (32)		14 (15)	1.17 (0.66 − 2.09)		1.83 (0.94 − 3.59)	
	Dar es Salaam	88 (13)		38 (13)		4 (11)	0.81 (0.39 − 1.69)		1.60 (0.63 − 4.01)	
	Verulam	180 (26)		70 (24)		24 (34)	3.08 (1.66 − 5.72)		2.21 (0.45 − 10.82)	
Sex at birth	Male	92 (13)		38 (13)		18 (47)	Ref	*p* < 0.001	Ref	0.006
	Female	605 (87)		252 (87)		45 (18)	0.20 (0.10 − 0.40)		0.29 (0.12 − 0.70)	
Categorised age	18−24	324 (46)		120 (41)		20 (17)	Ref	0.041	Ref	0.095
	25 or higher	373 (54)		170 (59)		43 (25)	1.60 (1.02 − 2.50)		1.53 (0.93 − 2.51)	
Marital status	Single	280 (40)		117 (40)		16 (14)	Ref	0.039	Ref	0.042
	Married/cohabiting/relationship	285 (41)		118 (41)		34 (29)	1.78 (1.09 − 2.92)		0.64 (0.32 − 1.30)	
	Divorced/separated/widowed	132 (19)		55 (19)		13 (24)	1.82 (0.98 − 3.36)		1.84 (0.92 − 3.70)	
Education	Primary or lower	281 (40)		117 (40)		25 (21)	Ref	0.933	Ref	0.013
	Secondary or higher	416 (60)		173 (60)		38 (22)	0.98 (0.63 − 1.54)		0.49 (0.28 − 0.86)	
Religion	Christian	536 (77)		226 (78)		55 (24)	Ref	0.233	Ref	0.774
	Muslim/other	161 (23)		64 (22)		8 (13)	0.73 (0.43−1.23)		1.11 (0.55 − 2.23)	
Rooms in house	3 or lower	555 (80)		227 (78)		43 (19)	Ref	0.006	Ref	0.165
	4 or higher	142 (20)		63 (22)		20 (32)	2.09 (1.23 − 3.52)		1.57 (0.83 − 2.96)	
Categorised occupation	Sex worker	414 (59)		176 (61)		26 (15)	Ref	*p* < 0.001	Ref	0.058
	Salon/bar/lodge workers	39 (6)		16 (6)		2 (13)	1.61 (0.62 − 4.19)		1.96 (0.68 − 5.64)	
	Subsistence fisheries workers	10 (1)		7 (2)		5 (71)	18.63 (4.10 − 84.70)		10.78 (1.76 − 66.12)	
	Unemployed	140 (20)		55 (19)		19 (35)	3.73 (2.08 − 6.69)		2.16 (0.60 − 7.76)	
	Other	94 (13)		36 (12)		11 (31)	1.47 (0.70 − 3.10)		1.13 (0.43 − 3.00)	
Reported risk behaviour as per last assessment prior to enrolment										
Number of partners (last 3 m)	5 or less 6 or higher	267 (38) 430 (62)		107 (37) 183 (63)		34 (32) 29 (16)	Ref 0.39 (0.24 − 0.63)	*p* < 0.001	Ref 0.86 (0.39 − 1.90)	0.708
Days since last condomless sex	2 or less 3 to 6 7 or higher	311 (45) 199 (29) 187 (27)		126 (43) 79 (27) 85 (29)		26 (21) 17 (22) 20 (24)	Ref 1.01 (0.59 − 1.73) 1.05 (0.62 − 1.78)	0.982	Ref 0.91(0.51 − 1.62) 0.77 (0.44 − 1.36)	0.669
Partners no condom (last 3 m)	2 or less 3 to 6 7 or higher	190 (27) 232 (33) 275 (39)		85 (29) 92 (32) 113 (39)		20 (24) 24 (26) 19 (17)	Ref 0.88 (0.50 − 1.54) 0.48 (0.28 − 0.83)	0.015	Ref 1.60 (0.80 − 3.17) 1.25 (0.59 − 2.67)	0.380
Transactional	No	171 (25)		69 (24)		27 (39)	Ref	*p* < 0.001	Ref	0.090
condomless sex	Yes	526 (75)		221 (76)		36 (16)	0.28 (0.17 − 0.48)		0.41 (0.14 − 1.15)	
Sex after recreational drugs (last 3 m)	No	617 (89)		256 (88)		53 (21)	Ref		Ref	0.676
	Yes	80 (11)		34 (12)		10 (29)	1.79 (0.91 − 3.54)	0.094	1.18 (0.55 − 2.52)	
Sex while drunk (last 3 m)	No	266 (38)		116 (40)		24 (21)	Ref		Ref	0.207
	Yes	431 (62)		174 (60)		39 (22)	1.29 (0.82 − 2.03)	0.262	1.37 (0.84 − 2.23)	
Any STI at baseline**	No	520 (75)		208 (72)		35 (17)	Ref	0.029	Ref	0.001
	Yes	177 (25)		82 (28)		28 (34)	3.56 (1.13 − 11.16)		2.46 (1.41− 4.28)	

¥ The outcome of interest for this analysis was adherence measured in DBS categorised as: Undetectable, <2 tablets per week, 2–3 tablets per week, 4–6 tablets per week, and daily dosing. § OR: crude odds ratios; aOR: adjusted odds ratio; aOR were adjusted for: site, sex, age, marital status, education, rooms in house, occupation, reporting transactional condomless sex, and having an STI at baseline.

* Column Percentages.

** Last results prior to trial enrolment. Participants were screened for Syphilis, Gonorrhoea, and Chlamydia. Some participants missed CTCT/NG testing at baseline due to test kit stock out.

**Table 4:** Prevalence of white coat dosing and associated factors associated among participants on TDF/FTC.

Overall	Categories	Participants with DBS results *n* (%)		White coat dosing				
				White coat dosing overall (%)	OR^§^ (95% CI)	*p* Value	aOR^§^ (95% CI)	*p* Value
		290 (41)		85 (29)				
Site	Masaka	88 (30)		16 (18)	Ref	0.002	Ref	0.050
	Mbeya	94 (32)		28 (30)	1.91 (0.95 − 3.84)		2.33 (0.99 − 5.48)	
	Dar es Salaam	38 (13)		9 (24)	1.40 (0.55 − 3.52)		0.57 (0.17− 1.90)	
	Verulam	70 (24)		32 (46)	3.79 (1.85 − 7.76)		3.08 (0.74 − 12.87)	
Sex at birth	Male	38 (13)		13 (34)	Ref	0.477	Ref	0.591
	Female	252 (87)		72 (29)	0.77 (0.37 − 1.59)		0.76 (0.27−2.11)	
Categorised age	18−24	120 (41)		33 (28)	Ref	0.569	Ref	0.729
	25 or higher	170 (59)		52 (31)	1.16 (0.69 − 1.95)		1.11 (0.62 − 1.96)	
Marital status	Single	117 (40)		32 (27)	Ref	0.670	Ref	0.555
	Married/cohabiting/relationship	118 (41)		38 (32)	1.26 (0.72 − 2.21)		0.61 (0.24 − 1.51)	
	Divorced/separated/widowed	55 (19)		15 (27)	0.10 (0.49 − 2.04)		0.94 (0.42 − 2.08)	
Education	Primary or lower	117 (40)		31 (27)	Ref	0.387	Ref	0.124
	Secondary or	173 (60)		54 (31)	1.26 (0.75 − 2.12)		0.58 (0.29 − 1.16)	
	higher							
Religion	Christian	226 (78)		62 (27)	Ref	0.189	Ref	0.005
	Muslim/other	64 (22)		23 (36)	1.48 (0.82 − 2.67)		3.52 (1.46 − 8.47)	
Rooms in house	3 or lower	227 (78)		60 (26)	Ref	0.043	Ref	0.484
	4 or higher	63 (22)		25 (40)	1.83 (1.02 − 3.29)		1.29 (0.63 − 2.65)	
Categorised	Sex worker	176 (61)		43 (24)	Ref	*p* < 0.001	Ref	0.037
occupation	Salon/ bar/lodge workers	16 (6)		8 (50)	3.09 (1.09 − 8.74)		3.95 (1.22 − 12.85)	
	Subsistence fisheries	7 (2)		0 (0)	−		−	
	Unemployed	55 (19)		28 (51)	3.21 (1.71 − 6.03)		2.39 (0.53 − 10.71)	
	Other	36 (12)		6 (17)	0.62 (0.24 − 1.59)		0.69 (0.19 − 2.47)	
Number of partners (last 3 m)	5 or less	107 (37)		39 (36)	Ref	0.042	Ref	
	6 or higher	183 (63)		46 (25)	0.59 (0.35 − 0.98)		1.16 (0.44 − 3.08)	0.763
Days since last condomless sex	2 or less	126 (43)		38 (30)	Ref	0.634	Ref	0.411
	3−6	79 (27)		20 (25)	0.79 (0.42 − 1.48)		0.66 (0.33 − 1.33)	
	7 or higher	85 (29)		27 (32)	1.08 (0.59 − 1.95)		1.05 (0.53 − 2.09)	
Transactional condomless sex	No	69 (24)		27 (39)	Ref	0.042	Ref	0.697
	Yes	221 (76)		58 (26)	0.55 (0.31 − 0.98)		1.29 (0.36 − 4.62)	
Sex after recreational drugs (last 3 m)	No	256 (88)		71 (28)	Ref	0.109	Ref	0.514
	Yes	34 (12)		14 (41)	1.82 (0.87 − 3.81)		1.35 (0.55 − 3.36)	
Sex while drunk (last 3 m)	No	116 (40)		27 (23)	Ref	0.066	Ref	0.050
	Yes	174 (60)		58 (33)	1.65 (0.97 − 2.81)		1.82 (1.00 − 3.30)	
Any STI at baseline*	No	208 (72)		66 (32)	Ref	0.151	Ref	0.296
	Yes	82 (28)		19 (23)	0.65 (0.36 − 1.17)		0.70 (0.36 − 1.36)	

% Col percentages.

* Last results prior to trial enrolment. Participants were screened for syphilis, Gonorrhoea, and chlamydia. some participants missed ct/nG testing at baseline due to test kit stock out.

§ OR: crude odds ratios; aOR: adjusted odds ratio aOR were adjusted for: site, sex, education, religion, occupation, and reporting sex while drunk (last 3 m) at baseline.

**Table 5:** Expected risk reduction in reference to previous trials.

TFV-DP levels in dried blood spots	% Observed adherence in a random sample (PrEPVacc)^§^			Estimated risk reduction as per the literature (95% CI)*			
	Week 8 (*N* = 290)	Week 26 (*N* = 39)		IPREX**	HPTN083***	HPTN084***	Assuming 85% protection if TFV-DP is detectable****
Below limit of quantification (BLQ)	24%	49%		0%	0%,	0%,	0%
< 350 fmol/punch ∼ (<2 tablets per week but detectable)	54%	38%		0%	49% (2-75)	16% (0-60)	85%
350−699 fmol/punch ∼ (2-3 tablets per week)	14%	13%		76%	99% (93-99)	80% (32-97)	85%
700−1250 fmol/punch ∼ (4-6 tablets per week)	7%	0%		96%	99% (98-99)	88% (43-99)	85%
>1250 fmol/punch ∼ (daily dosing)	1%	0%		99%	98% (93-99)	99% (0-99)	85%
Estimated risk reduction at week 8; week 26^¥^				18%; 10%	48%; 31%	27%; 16%	65%; 43%

§ Column percentages.

* Utilising data from multiple studies (including FEM-PrEP, VOICE, Partners PrEP, and HPTN082), Moore et al estimated that two, four and seven pills per week reduced HIV incidence (95% credible intervals) by 59.3% (29.9–95.8), 83.8% (51.7–99.8), and 95.9% (72.6–100)) among females [16]. These estimates have not been included here because they are comparable to those presented.

** IPREX was a placebo-controlled trial among males at high risk of HIV [4, 26]. (Risk reduction estimates from the IPREX trial have previously been used to infer the estimated risk reduction attributable to TDF/FTCTC adherence in the DISCOVER active-controlled trial secondary analyses by Glidden et al. [4,14]).

*** HPTN083 and HPTN084 [14] were active-controlled trials with a comparator TDF/FTCTC arm conducted among males at high risk of HIV, and females at high risk of HIV in sub-Saharan Africa respectively. The risk reduction estimates from the HPTN083 and 084 trials were inferred by contrasting incidence in higher adherence categories to the below limit of quantification (BLQ) category, hence are susceptible to confounding effects in the reference trials.

**** A systematic review of placebo controlled TDF/FTCTC trials concluded that participants with detectable levels of drug had 85% protection [17, 29].

¥ Assuming no confounding factors for the association between HIV incidence and PrEP adherence

## Data Availability

The datasets generated and analysed during this study are available from the corresponding author (SK) upon reasonable request.
