# Supplementary material for: Evaluating adherence in an active-controlled HIV pre-exposure prophylaxis trial (PrEPVacc) to inform the estimation of HIV incidence in a counterfactual placebo arm
Source: HIV Res Clin Pract. Author manuscript; Available in PMC 2026 Jul 24. (PMC7619267; doi:10.1080/25787489.2025.2513684)
Supplement: Table 1a,1b,1c,1d and Table 2 [file EMS216240-supplement-Table_1a_1b_1c_1d_and_Table_2.docx]

**Supplementary table 1a. Assessing associations of other adherence measures with TFV-DP levels in DBS**

|  |  | **As per TFV DP levels in RBCs (assessed using DBS) (last 6 weeks adherence)*^¥^*** | | | | |
| --- | --- | --- | --- | --- | --- | --- |
|  | **Participants with DBS results**  **n (%)** | **N (%) with adherence consistent with ≥ 2 pills/ week** | **OR(95% CI)^§^** | **P-value** | **aOR (95% CI)**** | **P-value** |
| **Self-reported adherence (before the last condomless sex act) (Of 5 sex acts)***** |  |  |  |  |  |  |
| 0 sex acts protected (or 0%) | 14 (5) | 0 (0) | 0.1 (0.03 – 0.32) | P<0.001 | 0.11 (0.03 – 0.43) | 0.018 |
| 1-2 sex acts protected (or >0% but <41%) | 20 (7) | 2 (10) | 0.50 (0.21 – 1.21) |  | 0.67 (0.25-1.78) |  |
| 3-4 sex acts protected(or >40% but <81%) | 96 (33) | 25 (26) | 0.92 (0.56 – 1.52) |  | 0.83 (0.48 – 1.42) |  |
| 5 sex acts protected (or >80%) | 160 (55) | 36 (23) | Ref |  | Ref |  |
| **Pill dispensing (6 months)** |  |  |  |  |  |  |
| MPR <75% | 27 (9) | 3 (11) | 0.24 (0.11 – 0.53) | P<0.001 | 0.26 (0.11 – 0.63) | 0.003 |
| MPR ≥75% | 263 (91) | 60 (23) | Ref |  | Ref |  |
| **Urine tests indicative of drug (last 2 days adherence) of 3 urine tests** |  |  |  |  |  |  |
| 0 tests (or 0%) | 46 (16) | 0 (0) | 0.02 (0.01- 0.04) | P<0.001 | 0.01 (0.00 - 0.02) | P<0.001 |
| 1 test (or >0% but <34%) | 31 (11) | 1 (3) | 0.17 (0.07 – 0.38) |  | 0.08 (0.03 – 0.21) |  |
| 2 tests ( or >33% but < 68%) | 63 (22) | 18(29) | 0.65 (0.35 – 1.19) |  | 0.59 (0.31 – 1.11) |  |
| 3 tests (or > 67%) | 150 (52) | 44 (29) | Ref |  | Ref |  |

***^¥^****The outcome of interest for this analysis was adherence measured in DBS categorised as: Undetectable, <2 tablets per week, 2-3 tablets per week, 4-6 tablets per week and daily dosing;* ***^§^****OR: Crude odds ratios; **aOR: Odds ratios adjusted for days since the last sex act, sex, site and other factors associated with adherence as per DBS; *Categories for participants with missing data or a missing result were determined using the available data e.g., if a participant had only two sex acts their data were presented as a proportion of two.*

**Supplementary table 1b. Assessing accuracy of urine tenofovir levels in reference to FTC-TP levels in DBS**

| **Urine Tenofovir test at visit 6 among those with DBS results** | **Recent adherence (FTC-TP) at visit 6** | | | | |
| --- | --- | --- | --- | --- | --- |
|  | **Undetectable** | **Unquantifiable but detectable** | | **Quantifiable (FTC-TP>0.1 pmol/punch)** | **Overall** |
| Test indicative of absence | 73 (89) | 4 (5) | | 5 (6) | 82 (100) |
| Test indicative of presence | 12 (6) | 52 (26) | | 138 (68) | 202 (100) |
| **Sensitivity**: Proportion of those with detectable drug (FTC-TP), whose urine tests were indicative of presence of drug.  $\frac{\left( 52+138 \right)*100}{(4+52+5+138)}=95\%$ | | | **Positive predictive value:** Proportion of those with urine tests indicative of presence of drug who have detectable drug (FTC-TP).  $\frac{\left( 52+138 \right)*100}{(12+52+138)}=94\%$ | | |
| **Specificity:** Proportion of those without detectable drug (FTC-TP), whose urine tests were indicative of absence of drug.  $\frac{\left( 73 \right)*100}{(73+12)}=86\%$ | | | **Negative predictive value**: Proportion of those with urine tests indicative of absence of drug who don’t have detectable drug (FTC-TP).  $\frac{\left( 73 \right)*100}{(73+4+5)}=89\%$ | | |

**6 participants with DBS results missed urine Tenofovir assessments at visit 6.*

**Supplementary table 1c. Comparing urine tenofovir levels to TFV-DP levels in DBS**

| **Urine Tenofovir test at visit 6 (measuring last 2 days adherence)** | **Long term adherence (TFV-DP) at visit 6** | | | | |
| --- | --- | --- | --- | --- | --- |
|  | **Undetectable** | **(<2 tablets per week)** | | **2 or more tablets** | **Overall** |
| Test indicative of absence | 51 (62) | 28 (34) | | 3 (4) | 82 (100) |
| Test indicative of presence | 15 (7) | 127 (63) | | 60 (30) | 202 (100) |
| **P1**: Proportion of those with detectable drug (TFV-DP), whose urine tests were indicative of presence of drug.  $\frac{\left( 127+60 \right)*100}{28+127+3+60}=86\%$ | | | **P3:** Proportion of those with urine tests indicative of presence of drug who have detectable drug (TFV-DP).  $\frac{\left( 127+60 \right)*100}{15+127+60}=93\%$ | | |
| **P2:** Proportion of those without detectable drug (TFV-DP), whose urine tests were indicative of absence of drug.  $\frac{\left( 51 \right)*100}{51+15}=77\%$ | | | **P4:** Proportion of those with urine tests indicative of absence of drug who don’t have detectable drug (TFV-DP).  $\frac{\left( 51 \right)*100}{51+28+3}=62\%$ | | |

**6 participants with DBS results missed urine Tenofovir assessments at visit 6.*

*- In table 1c we use proportions P1-P4, and not sensitivity, specificity, positive predictive value, negative predictive value, because urine tests and TFV-DP levels measure different aspects of adherence ie short-term Vs long-term.*

**Supplementary table 1d. Comparison of self-reported adherence (tablets taken before sex) with DBS results (FTC-TP and TFV-DP levels) at visit 6, stratified by the number of days since the last condomless sex act.**

|  | **Participants with DBS data (%)** | **Proportion who reported taking 2 pills before last sex act*** | **Proportion with detectable FTC-TP levels*** | **Proportion with quantifiable (>0.1 pmol/ punch) FTC-TP levels*** | **Proportion with TFV-DP levels indicating ≥ 2 pills per week*** |
| --- | --- | --- | --- | --- | --- |
| **Days since last condomless sex act at visit 6** | | | | | |
| Less than 3 days | 139 (48) | 84% | 66% | 49% | 22% |
| 3 - < 7 days | 80 (28) | 86% | 79% | 58% | 30% |
| >7days | 71 (24) | 79% | 62% | 41% | 13% |
| **Median days since last condomless sex act before visit 9**** | | | | | |
| Less than 3 days | 150 (52) | 86% | 67% | 46% | 22% |
| 3- < 7 days | 95 (33) | 83% | 73% | 56% | 28% |
| >7days | 45 (15) | 72% | 67% | 47% | 7% |

*Narrative: This table presents the varying proportions of participants adhering to PrEP, as per the different adherence measures, stratified by the number of days since their last condomless sex act. For example, among participants who reported their last sex act occurred <3days prior to a visit, 84% reported taking a pill before the sex act, 66% had detectable FTC-TP levels, 49% had quantifiable FTC-TP levels, and 22% had TFV-DP levels consistent with taking ≥ 2 pills per week.*

**At visit 6.*

***All assessments at visits 2-9 included to estimate a median for each participant.*

**Supplementary Table 2. Recent adherence vs average 6-week TDF/FTC adherence at visit 6 (week 8)**

| **FTC-TP (pmol/punch) at visit 6*** | **TFV-DP (fmol/punch) at visit 6**** | | | |
| --- | --- | --- | --- | --- |
|  | Undetectable | (<2 tablets per week) | 2 or more tablets | Overall |
| Undetectable | 61 (67) | 29 (32) | 1 (1) | 91 (100) |
| Unquantifiable but detectable | 9 (16) | 43 (77) | 4 (7) | 56 (100) |
| Quantifiable (FTC-TP>0.1 pmol/punch) | 0 (0) | 85 (59) | 58 (41) | 143 (100) |
| Overall | 70 (24) | 157 (54) | 63 (22) | 290 (100) |

*FTC-TP reflects adherence in the last 2-4 days; **TFV-DP reflects average adherence over the last 6 weeks.
